# Oilbirds produce echolocation signals beyond their best hearing range and adjust signal design to natural light conditions

**DOI:** 10.1098/rsos.170255

**Published:** 2017-05-24

**Authors:** Signe Brinkløv, Coen P. H. Elemans, John M. Ratcliffe

**Affiliations:** 1 Sound Communication and Behaviour Group, Department of Biology, University of Southern Denmark, 5230 Odense M, Denmark; 2 Department of Biology, University of Toronto Mississauga, Mississauga, Ontario, CanadaL5C 1C6

**Keywords:** biosonar, vision, multi-modal integration, convergent evolution, biophysical constraint

## Abstract

Oilbirds are active at night, foraging for fruits using keen olfaction and extremely light-sensitive eyes, and echolocate as they leave and return to their cavernous roosts. We recorded the echolocation behaviour of wild oilbirds using a multi-microphone array as they entered and exited their roosts under different natural light conditions. During echolocation, the birds produced click bursts (CBs) lasting less than 10 ms and consisting of a variable number (2–8) of clicks at 2–3 ms intervals. The CBs have a bandwidth of 7–23 kHz at −6 dB from signal peak frequency. We report on two unique characteristics of this avian echolocation system. First, oilbirds reduce both the energy and number of clicks in their CBs under conditions of clear, moonlit skies, compared with dark, moonless nights. Second, we document a frequency mismatch between the reported best frequency of oilbird hearing (approx. 2 kHz) and the bandwidth of their echolocation CBs. This unusual signal-to-sensory system mismatch probably reflects avian constraints on high-frequency hearing but may still allow oilbirds fine-scale, close-range detail resolution at the upper extreme (approx. 10 kHz) of their presumed hearing range. Alternatively, oilbirds, by an as-yet unknown mechanism, are able to hear frequencies higher than currently appreciated.

## 1. Background

Vision and echolocation allow animals that possess one or both systems to detect and localize objects well beyond their reach. Echolocation is an active auditory spatial sense and has independently evolved multiple times in vertebrate lineages [1]. Echolocators use three-dimensional auditory localization of objects in their surroundings and update their auditory scene as they move through space [2] with update rates reflected in the rate of signal production [3]. They gauge distance to objects based on the time elapsed between signal emission and echo return and, in laryngeal echolocating bats at least, determine elevation and azimuth from echo spectral cues and inter-aural time and intensity differences [2]. Object detection distance depends on signal intensity and frequency content, frequency sensitivity of the sonar receiver (ear) and the sizes of the objects themselves [4].

Echolocation has evolved in only a small subset of animals that operate under conditions of uncertain lighting. Laryngeal echolocating bats and toothed whales are the two groups most highly adapted for echolocation, and unlike many other predatory vertebrates, they do not have visual systems adapted specifically for long-range prey detection [5]. Benefitting from their high-frequency sensitive mammalian ear and specialized sound production systems, they manoeuvre and track prey using signals of partly or entirely ultrasonic content (greater than 20 kHz) that confer high resolution and are produced during pursuit at rates up to approximately 200 s^−1^ for bats [6] and approximately 600 s^−1^ for whales [7]. Laryngeal echolocating bats rely on echolocation as their primary spatial sense [8] and continuously adjust signal design and emission patterns in response to their surroundings, e.g. clutter conditions and conspecifics [9,10]. Toothed whales exhibit greater variation with respect to emission than do bats: some species probably always produce biosonar clicks when moving [11], while others do so only at great depths that are essentially devoid of light [12] or when actively foraging [13].

While it is unclear how many echolocating bat species have been considered, light level has only been reported to affect echolocation signal design or emission behaviour in a few species. Among laryngeal echolocators, *Megaderma lyra* (Megadermatidae) and the phyllostomids *Macrotus californicus* and *Phyllostomus discolor* reportedly emit fewer echolocation calls when flying in the laboratory under light levels approaching bright moonlight when compared with darkness [14–16]. However, at least for *M. lyra*, this reduction may be largely due to familiarity with the laboratory environment [17]. Recordings of tongue-clicking pteropodid bats (genus *Rousettus*) have produced similarly conflicting results [18–22], but recent work indicates that *Rousettus aegyptiacus* produces fewer clicks, of lower intensity, in the laboratory as light levels increase [22].

Only two groups of birds—the nocturnal oilbird *Steatornis caripensis* (Caprimulgiformes) and some diurnal swiftlets (Apodidae, *Aerodramus* and *Collocalia* spp.)—are known to echolocate, using syringeally produced signals [23,24]. Oilbirds and swiftlets both roost in caves and caverns, switching their sonar system on as they approach or emerge from their dark natural roosts and switching it off when visual input is more reliable [25]. Fenton [26] noted that mountain swiftlets (*Aerodramus hirundinacea*) ceased echolocating as they emerged from their cave roost into the brighter conditions of open sky. Similarly, captive oilbirds, dependent on echolocation to avoid the walls of a dark laboratory, reduced or stopped emitting echolocating signals when a light was turned on [27].

Oilbirds are guided by perhaps the most light-sensitive eyes among terrestrial vertebrates [28]. Such sensitivity is probably at the expense of visual acuity and colour perception [28,29]. Even for highly light-sensitive eyes, range and resolution decrease as light levels drop [28,30]. We therefore set out to explore to what extent oilbirds rely on echolocation under natural light conditions and, given conflicting past descriptions of oilbird echolocation signals [25], whether the birds are to any extent able to modify their echolocation emissions. We recorded oilbirds in the field at two colonies in Trinidad as they flew to and from their natural roosts during nightly foraging bouts and compared their echolocation behaviour under five different conditions. Our aim was to document whether these syringeal echolocators exhibit plasticity in biosonar signal design and emission rate under natural conditions, particularly given the presumed dominance of vision in dim light [28,29].

## 2. Material and methods

### 2.1. Recording sites

We used microphone arrays to record and position oilbirds as they echolocated while flying to and from two nesting caves in Trinidad. We recorded echolocation signals from oilbirds departing from their roost during four nights at Dunstan's Cave (Asa Wright Nature Center, Arima Valley), a partially roofed narrow section (approx. 15 m long and less than or equal to 3 m wide) of a river gorge. Upon emergence, the birds flew upstream towards a widening area of the gorge. Dunstan's Cave has a stable population of about 130 oilbirds nesting on ledges 3–5 m above the riverbed, which at the time of recording was coursing with fast-running water. During one of those four nights (8 February 2012), the Moon was full (97%) and hung above the gorge in a clear, cloudless sky. On the remaining three nights (25, 26, 28 January 2012), the Moon was absent or below 15% full. On two of these nights (25 and 28 January), we also recorded birds as they returned to the cave.

On 3 February 2012 under heavy cloud cover, we recorded another oilbird colony at Aripo Cave, a half-dome-shaped cavern without pronounced ambient water noise, embedded deep in undisturbed primary forest under a closed forest canopy (Aripo Valley). We recorded during opposite phases of the lunar cycle to allow comparison of relatively light and dark natural, ambient nocturnal light conditions. For each session, we used meteorological moon phase and forecasted cloud cover data supplemented by personal observations to select dates and estimate relative light levels at the recording sites (e.g. full moon, clear sky; new moon, cloud-covered sky). During recording sessions, we took notes on the behaviour of the recorded birds while observing them through a night vision scope (Night Owl Explorer NOCX3, JNL Trading Company, Aurora, IL, USA).

We recorded the birds as they flew under five natural conditions. In full moonlight, we recorded single birds exiting Dunstan's Cave without another bird within 10 m of them. On dark nights, we recorded single birds exiting and single birds entering Dunstan's Cave, and birds exiting Dunstan's Cave in proximity (within 5 m) of a conspecific. Also on a dark night, we recorded single birds from a second colony exiting a different cave roost (Aripo), which was a light-occluding site without water flowing through it.

### 2.2. Recording equipment

We recorded echolocation signals with a cross-shaped microphone array with two 0.5 inch free-field microphones (40AF with 26AH preamplifiers, G.R.A.S. Sound & Vibration, Holte, Denmark) at the centre and top position and six to eight 0.25 inch microphones (40BF with 26AC preamplifiers, G.R.A.S.) at the remaining positions, with 30 cm spacing in both the vertical and horizontal planes. Each microphone was calibrated (Sound Level Calibrator, type 4231, Brüel & Kjær Sound & Vibration Measurement A/S, Nærum, Denmark) and the signals amplified (+30 dB, UltraSoundGate power module, Avisoft Bioacoustics, Germany) prior to being digitized without filtering with 16-bit resolution (Avisoft UltraSoundGate 1216H). We initially sampled at 300 kHz per channel and later adjusted the sampling rate to 75 kHz per channel once we had verified the signal frequency content. Files initially sampled at rates greater than 75 kHz were down-sampled to an equal sampling size of 75 kHz prior to further analyses (Matlab, resample function).

### 2.3. Signal detection and three-dimensional positioning

**Figure 1.**
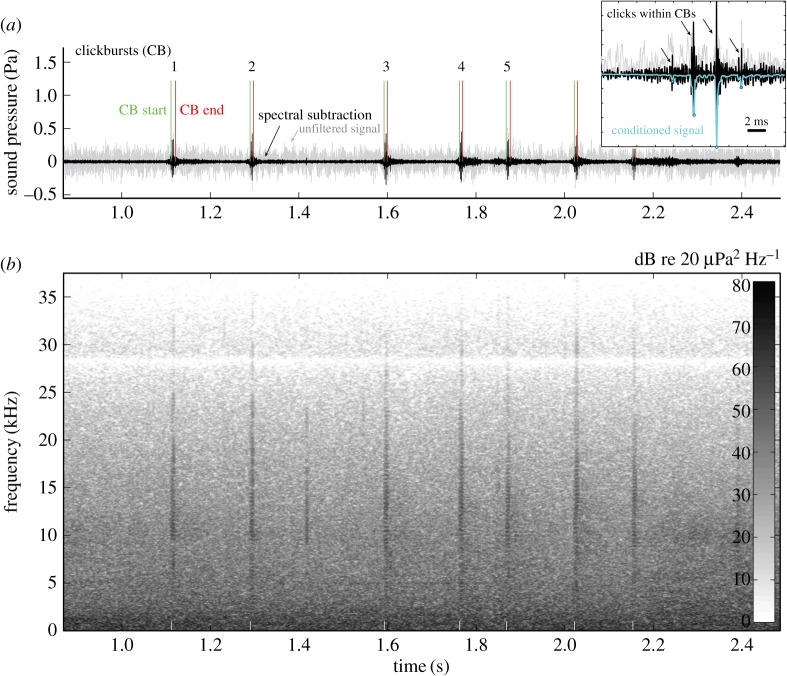
Oilbirds emit sequences of echolocation click bursts (CBs) in low-light conditions. (*a*) Oscillogram of a CB sequence emitted by an oilbird flying away from its roost. In the raw sound recordings, oilbird echolocation signals were often partly masked by ambient noise obscuring CBs and clicks within CBs (grey trace; raw sound recording). Applying a spectral subtraction algorithm (black trace; see Material and methods) facilitated CB extraction. Vertical lines indicate the start (green) and end (red) of each CB as detected by our automated CB extraction procedure. Inset in the upper right of (*a*) shows that each CB consists of multiple separate clicks. To find clicks within CBs, we calculated the product of two spectral subtraction methods ([35,36]; see Material and methods). Normalizing and integrating this product (conditioned signal, light blue trace) provided a robust signal to reliably extract click time stamps within CBs. (*b*) Spectrogram of CBs in (*a*) displaying sound pressure power spectra (grey scale, dB re 20 µPa^2^ Hz^−1^; FFT length: 1024, overlap: 1000, Hamming window).

We triangulated the position of oilbirds flying towards the array based on the arrival times of their echolocation signals at each microphone (Matlab script, see [31–34] for details). Oilbirds produced click bursts (CBs) each of which consisted of up to 8 individual clicks (figure 1). The loudest click within a CB was used as the time stamp to position the bird in three-dimensional space. A final dataset of 57 files was selected to include at least 10 flight sequences for each condition, based on five sequential, good signal-to-noise ratio CBs that were positioned in three-dimensional space.

To improve signal-to-noise ratio and facilitate objective detection of clicks heavily masked in low-frequency (e.g. wind and water) noise, we developed an automated analysis procedure to extract signal parameters. Briefly, each file was initially high-pass filtered (2 kHz, seventh-order Butterworth filter, filtfilt implementation to reduce phase distortion) (figure 1). We then applied a spectral subtraction algorithm (following Boll [35], implemented by Esfandiar Zavarehei) using the amplitude envelope calculated from the Hilbert transform of a 250 ms segment without CBs. The time stamps of CBs were localized reliably as peaks greater than 10% of the normalized Hilbert transform and greater than 50 ms interval from the previous CB. We isolated each CB by extracting 11 ms before and after the loudest click time stamp in the unfiltered recording, resulting in 22 ms (1650 points) segments. We also isolated a 50 ms noise segment prior to each time stamp.

To detect clicks severely masked by noise within each CB of the original recordings, we separately applied a multi-band spectral subtraction algorithm (following Kamath & Loizou [36], implemented by Esfandiar Zavarehei). We multiplied the multi-band and Boll spectral subtraction waveforms, then normalized and integrated the signal over 0.15 ms bins with a 0.10 ms sliding window. Individual clicks were isolated as local maxima above threshold (greater than 10% or 20 times the standard deviation of a 3 ms noise segment preceding each CB, whichever was lowest) using a click interval greater than 2 ms and click duration greater than 0.2 ms as criteria. We used these time stamps to isolate each click in the unfiltered recording (figure 1, inset). Automated localization of CBs and clicks was confirmed manually using Batsound v. 4.0 (Pettersson Electronik AB, Sweden).

### 2.4. Power spectral density and source level estimates

We calculated amplitude spectra of the extracted unfiltered CB and noise segments using the periodogram method. We estimated the source level of the CBs at 1 m by adding the transmission loss (TL) to the received level at the centre microphone, assuming a spherical spreading loss of 20 × log(*d*) and negligible atmospheric attenuation, given the distance (*d*) between the birds and our equipment. We used the same distance for each click within a CB, assuming displacement of the birds within CBs to be negligible at flight speeds less than 10 m s^−1^ (this study; see also [37–39]). We quantified received levels of clicks, which were highly transient and heavily masked in noise, as peak-to-peak values. We determined CB received levels by first applying a one-third octave band filter to mimic the response of a typical vertebrate cochlear hair cell and determining the RMS (root mean square) value of the entire isolated segment. We then applied one-third octave band filters at 2 and 8 kHz centre frequencies, corresponding, respectively, to where oilbird hearing is most sensitive and to the highest frequency for which their hearing sensitivity has been measured [40].

### 2.5. Signal detection distance estimates

We used the sonar equation [41], to estimate maximum target detection distances for the oilbirds for two target types, assuming target strengths of 0 dB for an extended background such as the birds' cave roost and −10 dB for a fruit 2–3 cm in diameter [32,42]. Assuming spherical spreading and using source levels estimated at a reference distance of 1 m, the sonar equation becomes DT = SL − 2(20log(DD)) − 2(ATT × (DD − 1 m) + TS), where DT is the detection threshold, SL the source level, ATT the atmospheric attenuation and DD the detection distance. Based on the oilbird audiogram [38], we used detection thresholds of 0 dB sound pressure level (SPL) (no noise) and +20 dB SPL (ambient noise, see [42]) for CB source levels extracted with the one-third octave band filter centred at 2 kHz and +30 dB SPL (no noise) and +50 dB SPL (ambient noise) for CB source levels extracted with the filter centred at 8 kHz. While ambient noise levels may be higher than estimated, only electrophysiological audiograms exist for oilbirds [40], such audiograms may be approximately 20 dB less sensitive than are behavioural techniques [43]. Detection distances were estimated by subtracting the two-way TL over the distance between the target and 1 m in front of the bird and adding target strength (TS, 0 for the cave, −10 dB for fruit [32,34,42]) to the source level, subsequently solving for detection distance by iteration until the correct detection threshold (0 or +30 dB SPL assuming no noise and +20 or +50 dB SPL assuming ambient noise) was reached. We assumed spherical spreading and used values for atmospheric attenuation calculated for 85% relative humidity and 25°C at 2 and 8 kHz, respectively.

### 2.6. Statistical analyses

All statistical tests were performed in JMP v. 12 (SAS Institute, Cary, NC, USA). All sound pressure data were used on a linear scale (Pa), rather than a log scale (dB), so as to not violate assumptions underlying our statistical tests. The mean values and 95% confidence intervals are reported as decibels (as per convention), but reflect conversions made to mean and 95% confidence intervals based on pascals and were made after statistical analyses had been run.

## 3. Results

Across all conditions, our recordings indicated that in the wild, oilbirds use CBs for echolocation that are less than 10 ms in duration and emitted at approximately five CBs per second (figure 1). Each CB typically contained two to five clicks (range 1–8; figure 1, inset) at 2–3 ms intervals and had a bandwidth of 7–23 kHz at −6 dB from signal peak frequency with emphasis at a plateau between 10 and 20 kHz (figure 1). All recordings were of birds flying 5–30 m from the cave entrance.

**Figure 2.**
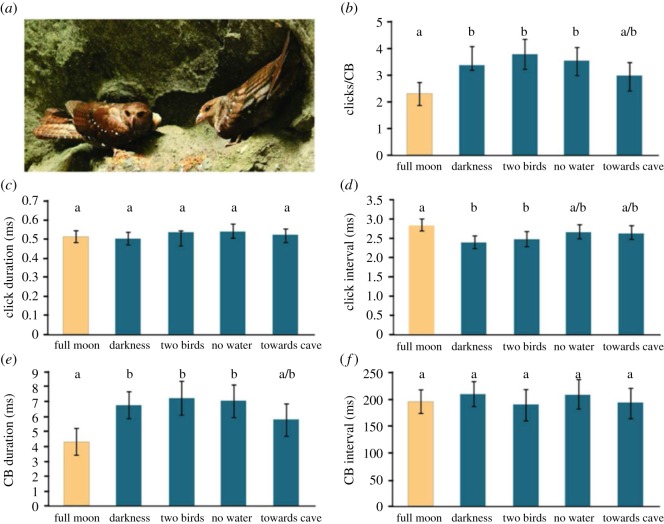
Temporal parameters of oilbird echolocation signals change with lighting condition. We recorded oilbird echolocation signals under five natural conditions, one in full moon, the other four on dark nights (either around new moon or in overcast conditions): (i) birds flying solo out of Dunstan's Cave in moonlight, (ii) birds flying solo out of Dunstan's Cave in darkness, (iii) birds flying with one or more conspecific out of Dunstan's Cave in darkness, (iv) birds flying solo out of Aripo Cave (no water) in darkness, and (v) birds flying solo into Dunstan's Cave in darkness. One-way ANOVAs and Tukey HSD *post hoc* tests were performed (*α *= 0.05 for all tests). Bars illustrate group means with 95% confidence intervals; groups that differed significantly from one another do not share the same letter (Photo: Roger Ahlman—www.pbase.com/ahlman).

We compared echolocation signal design, intensity and emission parameters across conditions (figure 2), using a minimum of 10 flight sequences per condition and five CBs per sequence (*N *= 285 CBs, parameters averaged for each bird/flight sequence). Specifically, we recorded oilbird echolocation signals under five natural conditions: (i) single birds exiting Dunstan's Cave in moonlight, (ii) single birds exiting Dunstan's Cave in darkness, (iii) birds exiting Dunstan's Cave in darkness in proximity of conspecifics, (iv) single birds exiting Aripo Cave in darkness, and (v) single birds entering Dunstan's Cave in darkness. Dunstan's Cave had a stream running through it; Aripo Cave did not.

Given that both colonies had more than 100 adult birds, that the birds seldom, if ever, flew one direction only to turn around again (as observed through infrared night scope), and that sequences selected were always recorded at least 3 min apart, we are confident that our analysed sample size reflects 57 birds and that our analyses do not suffer from pseudo-replication.

### 3.1. Comparison of temporal signal parameters across all conditions

We found that single birds exiting Dunstan's Cave in bright moonlight produced significantly fewer clicks per CB than birds exiting Dunstan's and Aripo Caves in darkness (ANOVA: *F*_4,52_ = 7.39, *p *< 0.0001; Tukey HSD *post hoc* tests; figure 2*b*). We found no significant differences in the duration of clicks produced by birds in any of the five conditions (ANOVA: *F*_4,52_ = 0.84, *p* = 0.51; figure 2*c*), but overall CB duration (i.e. time elapsed from beginning of first click to end of last click in a given CB) was significantly shorter in birds emerging in moonlight compared with birds flying away from Dunstan's and Aripo Caves in darkness (ANOVA: *F*_4,52_ = 6.4, *p *< 0.0003; figure 2*e*). Similarly, we found no significant condition-dependent differences in overall CB interval (ANOVA: *F*_4,52_ = 0.51, *p* = 0.73; figure 2*f*), but click interval within CBs was significantly longer in birds exiting Dunstan's Cave in moonlight than in birds exiting in darkness (ANOVA: *F*_4,52_ = 5.49, *p *< 0.0009; Tukey HSD *post hoc* tests; figure 2*d*).

### 3.2. Moonlight versus darkness: single birds exiting Dunstan's Cave

**Figure 3.**
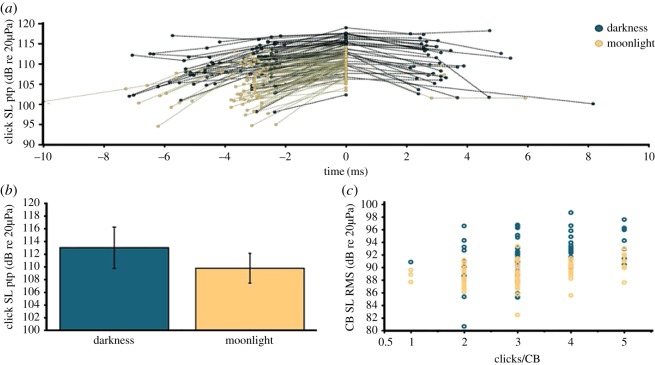
Source levels of oilbird echolocation signals change with lighting condition. (*a*) Illustrated crescendo and decrescendo of consecutive click source level (peak-to-peak, ptp) over time within each CB (aligned to the loudest click per CB at time = 0). Note that the loudest clicks appear towards the middle of a given CB. (*b*) The loudest clicks in darkness have a significantly higher source level than those produced in moonlight (mean ± 1 s.d.). (*c*) Source level of the entire CB (root-mean-square, RMS) increases significantly with number of clicks per CB in both light and dark conditions. See Results for further information on statistical analyses.

To further compare the birds' echolocation behaviour in moonlight with that on dark nights, we reconstructed their flight paths to calculate peak-to-peak source levels of clicks within CBs and RMS source levels for entire CBs for birds flying solo away from Dunstan's Cave either in full moonlight or in darkness (figure 3). Peak-to-peak source levels of clicks within CBs were significantly lower in moonlight than in darkness (two-sample *t*-test, *N* = 22, *t* = −5.75, *p *< 0.0001; figure 3*b*). CB RMS source levels were also significantly lower in moonlight compared with darkness and increased as the number of clicks per CB increased (two-way ANOVA: *F*_3,129_ = 42.56, *p *< 0.001; light condition: *t *= 11.1, *p *< 0.0001, clicks/CB: *t *< 0.002, *p *< 0.002, interaction: *t *= 1.3, *p*= 0.19; figure 3*c*). Within CBs with four or more clicks, click sound pressure levels typically increased and then decreased (figure 3*a*). Considered together, all measured parameters that differed between moonlight and darkness conditions (i.e. clicks per CB, CB duration, CB sound pressure level (SPL RMS), click SPL peak-to-peak) indicate that CBs produced in moonlight are of lower energy than the CBs produced in darkness (figures 2 and 3).

We estimated ecologically relevant detection distances of the cave and of a small (2–3 cm diameter) fruit using CB source levels from birds flying solo away from Dunstan's Cave in moonlight and in darkness. Our data show that in darkness at 2 kHz, the birds would detect cave walls as far away as 78 m at 0 dB (no noise) but only at 26 m at +20 dB (i.e. assuming ambient noise conditions). For the fruit, these values were 46 m and 15 m, respectively. At 8 kHz, detection values dropped to 16 m at +30 dB (no noise) and to 5.6 m at +50 dB (ambient noise) for a wall, and 10 m and 3.2 m for the fruit, respectively. In bright moonlight, where CBs are softer and shorter, at 2 kHz, these values were 56 m (no noise) and 19 m (ambient noise) for cave walls, and 33 m (no noise) and 11 m (ambient noise) for the fruit. At 8 kHz, they were 12 m (no noise) and 4 m (ambient noise) for cave walls, and 7 m (no noise) and 2 m (ambient noise) for fruit. These differences overall imply a 25% reduction in echolocation range in moonlight relative to darkness.

**Figure 4.**
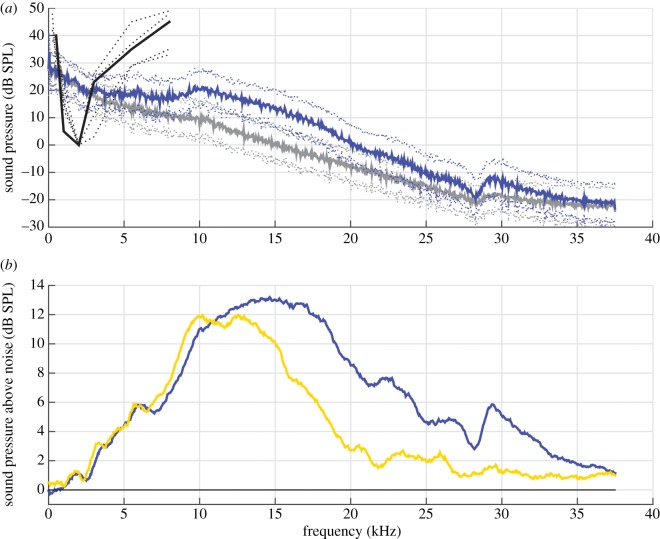
Spectral composition of oilbird echolocation signals misaligns with hearing range. (*a*) The sound amplitude spectrum (dB SPL) of 71 CBs (blue) and concurrent ambient noise segments (grey) show that CBs overcome ambient noise masking in a range from 2 to 35 kHz. The overlaid audiograms depict a best-frequency (BF) in oilbird hearing of approximately 2 kHz as measured by evoked potentials (range 1–4 kHz, +10 dB from BF; the black line depicts potentials from the inner ear, dashed black lines potentials from the forebrain nucleus, adapted from [40]). (*b*) Illustrates the spectral content of CBs in darkness and in moonlight after subtraction of ambient noise and using a relative dB range which is by convention set to zero for the lowest energy frequency components (blue; darkness (*N* = 71), yellow, moonlight (*N* = 65), bandwidth −6 dB from peak: darkness: 8–23 kHz; moonlight: 7–18 kHz). CBs therefore comprise an unexpected maximum energy plateau from 10 to 20 kHz at sound pressure levels approximately 10 dB higher than a CB local maxima at around approximately 2 kHz. Note, however, that the observed misalignment between plateau and the best frequency of oilbird hearing does not indicate oilbirds are deaf to their own echolocation signals, at least not at frequencies less than or equal to 8 kHz.

While CB frequency ranges in moonlight and darkness overlap considerably, we found that CBs produced in darkness not only contained more energy, but were also shifted slightly upwards in bandwidth (measured −6 dB from peak; moonlight: 7–18 kHz; darkness: 8–23 kHz; figure 4*b*).

## 4. Discussion

Our study provides evidence of echolocation signal modification correlated with light conditions in a bird biosonar system. The use of a multi-click CB as the functional signal design for biosonar is unique among echolocators (figure 1) and differs markedly from the tonal frequency modulated or constant frequency signals of laryngeal echolocating bats, but also from the signals produced by toothed whales, tongue-clicking rousette bats and echolocating swiftlets, which all use either single clicks or click doublets [1,5,20,25]. Our comparison of light and dark but otherwise equivalent conditions shows that oilbirds flying in the dark (at new moon) significantly reduced the interval between clicks within CBs, significantly increased the number of clicks per CB and significantly increased the peak-to-peak source level of their loudest clicks compared with birds flying in moonlight (figures 3 and 4). While the integration time of oilbird ears is not known, if we assume one typical of avian ears (approx. 100 ms [44]), the light-dependent signal adjustments we observed would collectively result in a change in perceived biosonar signal loudness. That is, all else being equal, oilbird CBs and returning echoes are louder in darkness than in moonlight because the birds produce more intense individual clicks and include more clicks per CB. In the same open space, this energy increase will impact the volume of space acoustically sampled. On the other hand, because we observed no temporal changes in CB production rate, unlike some bats [14–16,22], oilbirds do not appear to make light-dependent adjustments to sample update rate (figures 2 and 3).

Specifically, our results suggest that the total energy of a CB determines detection range. Source levels of CBs ranged from 81 to 100 dB SPL RMS at 1 m in all darkness conditions and 82 to 93 dB SPL RMS at 1 m in bright moonlight (figure 3*c*). Given these parameters, the CBs produced in moonlight would have resulted in about a 25% reduction in range when compared with those produced in darkness (e.g. a detection reduction from 26 to 19 m for cave walls under natural ambient conditions). We posit that in bright moonlight, oilbirds rely on vision for medium- to long-range object detection and use echolocation to supplement vision for more accurate short-range distance detection and feature resolution of small objects. Less energy is probably put into echolocation signal production in moonlight as the return on investment is reduced at greater light levels that allow for better long-range vision [45]. We therefore hypothesize that in darkness, oilbirds use echolocation alongside vision for short- to medium-range spatial orientation, as echo information reinforces or reveals objects at distances not easily discerned using vision alone. Conversely, across all conditions considered, oilbirds did not change the rate at which they produced CBs (approx. 5 CBs per second; figures 1 and 2*f*). Thus, regardless of ambient light or noise level, proximity to conspecifics, or flight direction towards or away from their roost, the birds sampled their environment through echolocation at the same rate. Furthermore, other than the changes to CB design in moonlight (figure 2*b*), we observed no significant changes in echolocation due to the presence of conspecifics or background noise from the stream running through Dunstan's Cave (figure 2).

The observed relationship between CB source level and clicks per CB (figures 1 and 3) provides clues about the mechanism by which oilbirds control the source level of each click and CB. Assuming a myoelastic-aerodynamic sound source (as found in other birds; see [46]), the vocal folds in the bird's syrinx should exhibit self-sustained oscillations above a threshold pressure, while sound excitation occurs by the sudden start or stop of airflow as the folds open and close [46]. In oilbirds, each click may result from a single acoustic excitation of the upper vocal tract by a single opening or closing of the vocal folds. So long as the subsyringeal pressure is above phonation threshold, clicks would then be produced at a consistent rate, consistent with the stable click interval within each CB. As subsyringeal pressure increases over the course of a CB's emission [23], source levels of subsequent clicks increase as aerodynamic forces increase (figures 1 and 3*a*), leading to higher velocity and sharper excitation of the vocal tract. The mechanism for increasing CB source level may thus be increasing of subsyringeal pressure during CBs. However, these hypotheses regarding sound production physiology remain to be tested.

Our results corroborate previous indications that oilbirds possess a relatively simple echolocation system (reviewed in [25]), complementing vision rather than substituting for it under dark night sky conditions. However, oilbirds' unique signal design allows for adaptive control of CB energy input and output. Intriguingly, the stereotyped CB emissions of approximately five CBs per second across conditions roughly matches our estimate of the birds' wing beat rate per second (see also [37–39]; figures 1 and 2*f*), an impression corroborated by biomechanical models for birds of oilbird size and wingspan [47]. When in transit from roost to foraging grounds, laryngeal echolocating vespertilionid bats tend to produce one echolocation signal per wing beat cycle [48], a coupling thought to reduce and perhaps even eliminate the energetic costs of in-transit signal production [49,50]. Whether something similar is happening in oilbirds is unknown.

The overall structure of the birds' echolocation signals (clicks per CB; CB duration, CB frequency spectra; figures 1, 2 and 4) we document here is close to that reported by Griffin [27]. This emphasizes that the longer, lower frequency signals described in later studies [23,40] may have been squawks (alarm calls) and not echolocation signals, a possibility acknowledged by Suthers & Hector [23], as in both studies, birds were recorded while being handled or as they hovered in cages. Notably, we found CBs to have most energy at 10–20 kHz. While this range is only slightly higher than the 6–10 kHz reported by Griffin [27], it is well above the range given by Konishi & Knudsen [40] who suggested that oilbirds echolocate with signals of most energy matched to their area of best hearing, i.e. at 2 kHz. The difference in our findings compared with past results may also be a result of the technologies and computation methods, which were not available for the early studies.

No bird is known to hear sounds of frequencies much above 10 kHz [50,51]. The much higher-frequency sensitivity of most mammals' hearing results from presumably independently evolved specializations of the inner and middle ear [52], allowing for the use of high-frequency (greater than 80 kHz) echolocation signals by bats [53] and of communication signals in non-echolocating mammalian groups (e.g. rodents, primates) [54–56]. High frequencies (and hence, short wavelengths) provide echoes from small objects and fine structural resolution of larger ones. For example, in the laboratory, the big brown bat, *Eptesicus fuscus*, uses frequency-modulated echolocation signals, which sweep from 100 to 30 kHz to detect wires as thin as 100 µm and discriminates size differences of as little as 50 µm [2,4,57]. Notably, however, echolocation signals of lower peak frequencies are not unheard of in mammals, as several laryngeal echolocating bats use signals with peak frequency of approximately 10 kHz to effectively detect flying insect prey (e.g. the vespertilionid, *Euderma maculatum* uses a frequency of approximately 9 kHz [58]; the molossid, *Tadarida teniotis*, uses a frequency of approximately 11 kHz [59]). Based on our current understanding of how frequency affects minimum size detection in echolocation, oilbirds should be able to detect objects smaller than 2 cm at 8 kHz. However, oilbirds can only reliably avoid discs of 20 cm diameter or greater in complete darkness [27,40].

The presented mismatch between the best frequency sensitivity in the oilbird ear (approx. 2 kHz, see [40]) and the frequency of maximum energy (10–20 kHz) in their echolocation signals (figure 4) is exceptional among echolocators and eared animals in general, as ears are typically broadly tuned to the most relevant signals for a given species (e.g. echolocation signals in bats, and mating calls, offspring distress calls and predator cues in non-echolocators, including other birds). For oilbirds, this mismatch probably reflects high-frequency constraints of the avian ear (i.e. lack of sensitivity above 10 kHz), but future work could reveal that oilbirds are exceptional among birds. We note, however, that (i) oilbirds' ears are well matched to the frequencies of their assumed social signals [23], and (ii) even at 2 kHz, our results indicate CB echoes returning from perpendicular cave walls greater than 20 m away would be audible to oilbirds under natural ambient conditions.

We speculate, however, that while this mismatch is most likely real, it may reflect a functional compromise. The syrinx is not constrained to producing signals only within the range of avian best hearing (2–5 kHz), as evidenced by the seet calls of songbirds (greater than or equal to 8 kHz) that are used as short-range alarm calls that are difficult for predatory birds to detect or localize [60]. The echolocation signals of oilbirds are also apparently specialized for short-range transmission, in this case auto-communication. By emphasizing frequencies at the maximum range of the oilbird's hearing sensitivity (rather than their best frequencies; figure 4), we hypothesize that oilbirds may be maximizing the resolving power of their sonar system for close-range representations of cliff and cave walls and precision perching in dark roosts while avoiding small branches and other obstacles in flight. Spectral content of the signal greater than or equal to 5 kHz would, even with less sensitive hearing at these frequencies, also provide some masking release in surroundings of high ambient noise (e.g. tropical forest and watercourses), because for such noise energy is typically concentrated below 5 kHz, particularly at close range when echoes will be perceived as being louder. This hypothesis waits to be tested but would—at least partially—account for the energy oilbirds put into frequencies above maximum sensitivity.

While the inclusion of echolocation for the multimodal (i.e. visual, olfactory and tactile) detection of the oilbird's preferred foods (oil palm fruit and tropical laurels [37,38,61]) has not been observed [25], at close range their echolocation signals could reflect potentially useful information about position and size of conspicuous fruits, as well as obstacles between themselves and the plants. Research at oilbird foraging sites seems warranted, as anecdotal reports suggest that birds do vocalize away from their roosts [38], but, to our knowledge, these sounds have never been recorded. Further study of their auditory capabilities and signal production mechanisms would also prove illuminating. Overall, our results suggest that oilbirds do not, as previously suggested, match their biosonar emissions to hearing ability and, as shown in a handful of bat species under controlled conditions, oilbirds do adjust the intensity of their unique multi-unit signals to light levels while flying in the field. Hence, oilbird echolocation may be more impressive and unusual than is currently appreciated and renewed attention to the interaction of vision and echolocation in this and other echolocating birds will be instructive.

## Data accessibility

Our data are deposited at Dryad: http://dx.doi.org/10.5061/dryad.fq068 [62].
